# Occupational Benzene Exposure and Lymphoma Risks: Vlaanderen et al. Respond

**DOI:** 10.1289/ehp.1104167R

**Published:** 2011-11-01

**Authors:** Jelle Vlaanderen, Qing Lan, Nat Rothman, Hans Kromhout, Roel Vermeulen

**Affiliations:** Section of Environment and Radiation, International Agency for Research on Cancer, Lyon, France; Division of Cancer Epidemiology and Genetics, National Cancer Institute, National Institutes of Health, Department of Health and Human Services, Bethesda, Maryland; Institute for Risk Assessment Sciences, Utrecht University, Utrecht, the Netherlands, E-mail: R.C.H.Vermeulen@uu.nl

We appreciate Sorahan’s interest in our study (Vlaanderen et al. 2011). We first evaluated the article by Sorahan et al. (2005) for inclusion in our meta-analysis based on its analysis of cancer incidence, which is consistent with our stated preference for using incidence rather than mortality data when both were available (Vlaanderen et al. 2011). Because the authors themselves had expressed serious concerns with regard to the underascertainment of cancer registrations (incidence) (Sorahan et al. 2005), we decided not to include these data and instead considered their mortality analysis, which was included in the same article (Sorahan et al. 2005). We then decided to exclude their mortality data as well because of their “inability to identify the type of cancer for a number of cancer deaths” (Vlaanderen et al. 2011). A total of 9% of all cancer deaths were not identified by type by Sorahan et al. (2005), compared to 2–6% from the publications we considered for inclusion that provided such data (9 of 40 cohorts reviewed). We did not make this decision based on the SMR for this category, as Sorahan claimed in his letter. Inclusion of the mortality data from Sorahan et al. (2005) has a negligible impact on our results [Table 1 compared with Supplemental Material, Table 1 of our paper (http://dx.doi.org/10.1289/ehp.1002318)] and does not alter our conclusion that this meta-analysis provides support for an association between occupational exposure to benzene and increased risk of multiple myeloma, acute lymphocytic leukemia, and chronic lymphocytic leukemia (Vlaanderen et al. 2011).

**Table 1:** Pooled risk estimates (and 95% confidence intervals) for AML and five lymphoma subtypes stratified by start of follow-up and AML significance level and including data from Sorahan et al. (2005).

Lymphoma subtype/AML significance level*^a^*		No. of studies	No. of cases	Meta relative risk (all studies)	No. of studies	No. of cases	Meta risk ratio (start follow-up before 1970)	No. of studies	No. of cases	Meta risk ratio (start follow-up 1970 and later)
AML												
A–E (all studies)		22	229	1.69 (1.38–2.08)*		13	131	1.47 (1.12–1.92)*		9	98	2.08 (1.59–2.72)
A–D		22	229	1.69 (1.38–2.08)*		13	131	1.47 (1.12–1.92)*		9	98	2.08 (1.59–2.72)
A–C		17	204	1.87 (1.57–2.22)		9	112	1.72 (1.38–2.15)		8	92	2.11 (1.61–2.77)
A–B		12	144	2.15 (1.76–2.63)		6	76	1.99 (1.51–2.60)		6	68	2.41 (1.77–3.29)
A		10	120	2.38 (1.89–2.99)		5	63	2.13 (1.57–2.89)		5	57	2.88 (1.95–3.99)
HL												
A–E (all studies)		28	149	1.00 (0.84–1.18)		20	126	1.01 (0.84–1.23)		8	23	0.91 (0.59–1.40)
A–D		13	72	0.99 (0.78–1.27)		9	61	1.03 (0.79–1.35)		4	11	0.83 (0.47–1.48)
A–C		10	42	0.84 (0.61–1.16)		6	31	0.84 (0.57–1.24)		4	11	0.83 (0.47–1.48)
A–B		6	10	0.57 (0.30–1.10)		3	9	0.65 (0.31–1.38)		3	1b	0.40 (0.11–1.44)
A		5	10	0.61 (0.31–2.19)		3	9	0.65 (0.31–1.38)		2	1c	0.46 (0.10–2.09)
NHLd												
A–E (all studies)		34	662	1.00 (0.89–1.12)*		23	467	0.93 (0.82–1.05)		11	195	1.21 (0.94–1.55)*
A–D		16	398	0.96 (0.81–1.14)		9	223	0.83 (0.68–1.01)		7	175	1.18 (0.91–1.53)*
A–C		14	359	0.98 (0.81–1.18)		7	184	0.83 (0.65–1.05)		7	175	1.18 (0.91–1.53)*
A–B		8	145	1.16 (0.85–1.57)		3	55	0.89 (0.62–1.27)		5	90	1.38 (0.92–2.06)*
A		7	116	1.10 (0.78–1.55)		3	55	0.89 (0.62–1.27)		4	61	1.40 (0.79–2.51)*
MM												
A–E (all studies)		27	290	1.11 (0.97–1.26)		17	210	1.06 (0.92–1.22)		10	80	1.26 (0.92–1.71)
A–D		15	166	1.13 (0.93–1.37)		8	111	1.06 (0.87–1.30)		7	55	1.27 (0.81–2.00)*
A–C		13	143	1.15 (0.91–1.44)		6	88	1.08 (0.86–1.34)		7	55	1.27 (0.81–2.00)*
A–B		8	75	1.40 (1.02–1.90)		3	35	1.20 (0.73–2.00)		5	40	1.58 (1.03–2.44)
A		7	62	1.42 (0.97–2.08)		3	35	1.20 (0.73–2.00)		4	27	1.75 (0.94–3.26)
ALL												
A–E (all studies)		18	47	1.41 (1.02–1.97)		11	30	1.27 (0.86–1.87)		7	17	1.92 (1.00–3.67)
A–D		18	47	1.41 (1.02–1.97)		11	30	1.27 (0.86–1.87)		7	17	1.92 (1.00–3.67)
A–C		12	29	1.36 (0.88–2.10)		6	15	1.04 (0.60–1.81)		6	14	2.10 (1.04–4.25)
A–B		8	16	1.59 (0.85–2.99)		3	5	0.98 (0.38–2.58)		5	11	2.28 (0.99–5.26)
A		6	12	1.52 (0.71–3.26)		2	3	0.88 (0.27–2.81)		4	9	2.30 (0.84–6.29)
CLL												
A–E (all studies)		19	116	1.16 (0.81–1.65)*		12	74	0.91 (0.56–1.48)*		7	42	1.63 (1.09–2.44)
A–D		19	116	1.16 (0.81–1.65)*		12	74	0.91 (0.56–1.48)		7	42	1.63 (1.09–2.44)
A–C		14	98	1.20 (0.78–1.84)*		8	60	0.91 (0.47–1.75)		6	38	1.61 (1.00–2.59)
A–B		9	62	1.37 (0.80–2.35)*		5	43	1.13 (0.43–2.97)		4	19	1.84 (1.12–3.02)
A		7	50	1.36 (0.74–2.51)		4	41	1.40 (0.49–4.01)		3	9	1.33 (0.64–2.76)
Abbreviations: ALL, acute lymphocytic leukemia; AML, acute myelogenous leukemia; CLL, chronic lymphocytic leukemia; HL, Hodgkin lymphoma; MM, multiple myeloma; NHL, non-Hodgkin lymphoma. Data presented here correspond to Supplemental Material, Table 1 from Vlaanderen et al. (2011; http://dx.doi.org/10.1289/ehp.1002318). Sorahan et al. (2005) was categorized as follow-up starting before 1970; AML significance level A [relative risk > 1 (p < 0.1)]; exposure assessment quality D (qualitative indication that benzene exposure had occurred). No observed cases were reported for ALL by Sorahan et al. We therefore calculated continuity-corrected relative risks (observed and expected number of cases + 1) and estimated associated 95% confidence intervals with mid-P exact. Values that are different from those in the original analyses are in italic type. aAML significance level categories: A, AML risk estimate > 1 (p < 0.1); B, AML risk estimate > 1 (p < 0.2); C, AML risk estimate > 1 (p > 0.2); D, AML risk estimate reported; E, AML risk estimate not reported. bTwo of three studies reported null cases (continuity correction was applied in the meta-analysis). cOne of two studies reported null cases (continuity correction was applied in the meta-analysis). dNHL or lymphosarcoma/reticulosarcoma (preferred NHL if the study reported both). *p < 0.1 for between-study heterogeneity.												
